# Competence required while caring for people living with mental illness in the ambulance care setting: a Delphi study

**DOI:** 10.1007/s44192-025-00140-6

**Published:** 2025-02-21

**Authors:** Mats Holmberg, Staffan Hammarbäck, Henrik Andersson

**Affiliations:** 1 Faculty of Health and Life Sciences, Department of Health and Caring Sciences, Linnaeus University, Växjö, Sweden; 2 Centre of Interprofessional Collaboration Within Emergency Care (CICE), Linnaeus University, Växjö, Sweden; 3 Centre for Clinical Research Sörmland, Uppsala University, Eskilstuna, Sweden; 4 Department of Ambulance Service, Region Sörmland, Katrineholm, Sweden; 5 Faculty of Caring Science, Work Life and Social Welfare, University of Borås, Borås, Sweden; 6 PreHospen – Centre for Prehospital Research, University of Borås, Borås, Sweden

## Abstract

**Background:**

People living with mental illness form a significant component of individuals presenting to emergency care services. Ambulance care embraces the care and treatment given to people of all ages who have suffered a sudden illness or injury and is carried out twenty-four-seven, regardless of setting and organizational belonging.

**Aim:**

The aim was to explore ambulance clinicians’ competence requirements in caring for people living with mental illness.

**Method:**

The study had a deductive and explorative design. A Delphi method was adopted using a group of experienced individuals recruited from the emergency care chain and non-governmental organizations (N = 15). An initial open-ended questionnaire was distributed covering three questions about; (1) knowledge, (2) skills and (3) attitudes that ambulance clinicians need to care for people living with mental illness. The informants’ answers were analysed using a manifest content analysis ending up in statements designed into a questionnaire that was sent out digitally in two rounds.

**Results:**

The 57 statements that reached consensus could be categorised as referring to knowledge (n = 26), skills (n = 13) and attitude (n = 18).

**Conclusion:**

Ambulance clinicians are expected to manage a range of incidents involving people living with mental illness, demanding knowledge of mental illness and the skills of mental health assessment, to ensure ambulance clinicians have the ability and non-judgmental attitude to make appropriate decisions within a caring encounter.

**Supplementary Information:**

The online version contains supplementary material available at 10.1007/s44192-025-00140-6.

## Introduction

Ambulance clinicians have an important role in caring for people living with mental illness. The number of people living with mental illness is increasing and is a global public health problem affecting millions of individuals [1, 2]. Research indicates that people living with mental illness are a sizeable group that presents to emergency care services [1, 3–6]. Emergency care embraces the care and treatment given to people of all ages who have suffered a sudden illness or injury and is carried out twenty-four-seven, regardless of setting and organizational belonging [7]. Emergency care can be viewed as an interconnected emergency care chain that consists of different actors who cooperate and collaborate with each other in people's care [8]. Actors in emergency care services include clinicians from community health and medical care, primary care services, ambulance services and acute or psychiatric emergency departments. Research also indicates that people living with mental illness do not always have access to professional emergency care facilities [9–12]. In some studies, people expressed feelings of stigmatization, humiliation, and discrimination in the care provided [13–15]. Such negative attitudes should be considered serious obstacles to successful emergency healthcare and influence individuals' opportunities for a good daily life, good quality of life, and a good balance of life’s opportunities and challenges.

In this study, we discuss emergency care in relation to ambulance services only. The evolution of ambulance services has brought a greater focus on assessing, treating, and helping people to attend the correct branch of emergency care services [16]. Historically, the ambulance service has conveyed people with different care needs to acute emergency departments [17, 18]. The collaboration between ambulance services and mental health services has been inadequate, which probably has resulted in negative outcomes for people living with mental illness [19]. It is important to define what competence means in relation to caring for people living with mental illness in the ambulance care setting, whether or not they seek ambulance care for primary mental health issues. In this study, competence is viewed as the individual capacity to handle specific work-related situations [20], and the requirements for that competence are influenced by, for example, colleagues and interprofessional teams [21]. Competence is also viewed as a clinical proficiency to apply one’s knowledge, skills, and attitudes in specific work-related situations [22]. Ambulance care is provided on a face-to-face personal level thus the view of the individual and the individual's opportunities and resources are realized [23]. Collaboration between different professionals is a prerequisite for creating patient-safety in the ambulance services [24]. Historically, ambulance clinicians have better competence to manage people with physical care needs rather than mental health needs [25, 26]. Consequently, there is a risk that people living with mental illness are not assessed correctly, that only physical care needs are attended to or that individuals are referred to the wrong care setting. Therefore, it is important to examine what competence is required while caring for people living with mental illness, to ensure a broad and multifaceted competence of the ambulance clinicians which is needed to assess and care for people living with mental illness and manage their mental health care needs. This applies regardless of whether individuals seek care for their mental health specifically or experience physical symptoms primarily or secondarily due to their mental health condition. In such cases, ambulance clinicians must adopt a holistic perspective on the patient's symptoms and overall health, recognizing that the physical, psychological, and existential aspects of suffering are interconnected [27]. Previous research has pointed to the need for competence development for ambulance clinicians when managing people living with mental illness [28–30]. However, the concept of mental illness includes many different conditions and there is a gap in the current research field as to what aspects of competence that most prominently needs to be developed.

## Aim

The aim was to explore ambulance clinicians’ competence requirements in caring for people living with mental illness.

## Materials and methods

### Design

To achieve this aim, a deductive explorative design incorporating a classic Delphi method was adopted [31]. The Delphi method is grounded in the assumption that a group of experts’ joint opinion is more valid than individual opinions. Deductively, Eraut's [21] definition of competence (which encompasses the concepts of knowledge, skills, and attitudes) was used to formulate questions and analyze data. The study followed the SRQR-checklist [32] (see Supplementary file 1).

### Expert panel

The experts were identified and selected by the authors in collaboration with four ambulance nurses working with the development of mental health care in an ambulance service in eastern Sweden. The ambulance nurses had an extensive network of colleagues with expert knowledge from different perspectives on care for people living with mental illness. The selection process started with an overview of potential experts’ perspectives was developed into a mind map. Experts from the emergency care chain and non-governmental organisations were considered relevant for the study. The mind map was revised by the authors and the four ambulance nurses, during two sessions, to assess the experts’ different experiences from prehospital care and care of people with mental illness and how these experiences correlated to the study’s aim. A final group of eighteen experts (N = 18) were agreed upon. The variety within the expert group was expected to generate different perspectives on competence in 1) caring for patients with mental illness and 2) ambulance care. The experts were contacted via e-mail with written information of the study together with an informed consent form. All experts gave their consent to participate in a responding e-mail. However, some did not respond in the first round giving an enrolled expert group of 15 informants (see Table 1).

**Table 1:** Overview of informants' demographic characteristics (Round 1 to 3)

	Round 1	Round 2	Round 3
Gender			
Male	7	7	7
Female	8	7	6
Organisations represented by the study's informants			
Hospital mental health care	6	5	5
*Adult mental health care*	*4*	*4*	*4*
*Mental health care*	*1*	*0*	*0*
*Addiction care*	*1*	*1*	*1*
Somatic care	6	6	5
*Ambulance care*	*4*	*4*	*4*
*Primary care*	*1*	*1*	*1*
*Municipal care*	*1*	*1*	*0*
Non-governmental organisations	3	3	3
*Patients’ association*	*1*	*1*	*1*
*Relatives' association*	*1*	*1*	*1*
*Church*	*1*	*1*	*1*
Role/title			
Coordinator patient influence	1	1	1
Registered nurse (ambulance nurse, mental health nurse, community nurse)	6	6	5
Care assistant (mental health, emergency medical technician)	3	3	3
Manager	2	1	1
Deacon	1	1	1
Educator	1	1	1
Researcher	1	1	1
Education (each informant can have multiple qualifications)			
Nurse Assistant	7	7	7
Medical doctor (specialist in general medicine and psychiatry)	1	0	0
Registered nurse without specialist education	1	1	1
Registered nurse with specialist education in mental health or ambulance care	6	6	5
Deacon education	1	1	1
Research education	1	1	1
Care assistant	1	1	1

### Data collection and analysis

Data collection and analysis were undertaken between the 22nd of April and 30th of August 2022.

#### Round 1

An open-ended questionnaire was distributed via e-mail, covering three questions; (1) What knowledge—according to your experience—does ambulance clinicians need to care for people living with mental illness? (2) What skills—according to your experience—do ambulance clinicians need to care for people living with mental illness? (3) What attitudes—according to your experience—do ambulance clinicians need to care for people living with mental illness? Using free-text answers, the data from the fifteen informants were analysed using manifest content analysis [33]. The analysis was carried out by the first and third author and the second author reviewed the analysis. To minimise bias during the analysis, the authors reflected on their preconceptions, arising from different clinical backgrounds of the authors, being aware that there was a risk that it could have impacted the analysis. The first author has e.g., a clinical and research background in ambulance care, the second author has his background e.g., as clinical ambulance nurse and doctoral student researching patients with suicidality in ambulance care, and the third author e.g., has clinical background as an emergency nurse and researcher in competence development. Subsequently, the statements were reformulated for clarification, with the intent of remaining close to the concepts and words used by the informants and reducing the impact of the authors’ misconceptions. Statements were compared with each other to reduce redundancy and sorted into the three pre-defined categories (knowledge, skills and attitudes). Finally, the analysis resulted in 58 statements incorporated into a questionnaire using an online survey tool (SurveyMonkey®). Each statement was accompanied with a five-point Likert scale covering; 1) Disagree, 2) Somewhat disagree, 3) Neutral, 4) Somewhat agree and 5) Strongly agree.

#### Round 2

**Fig.1:**
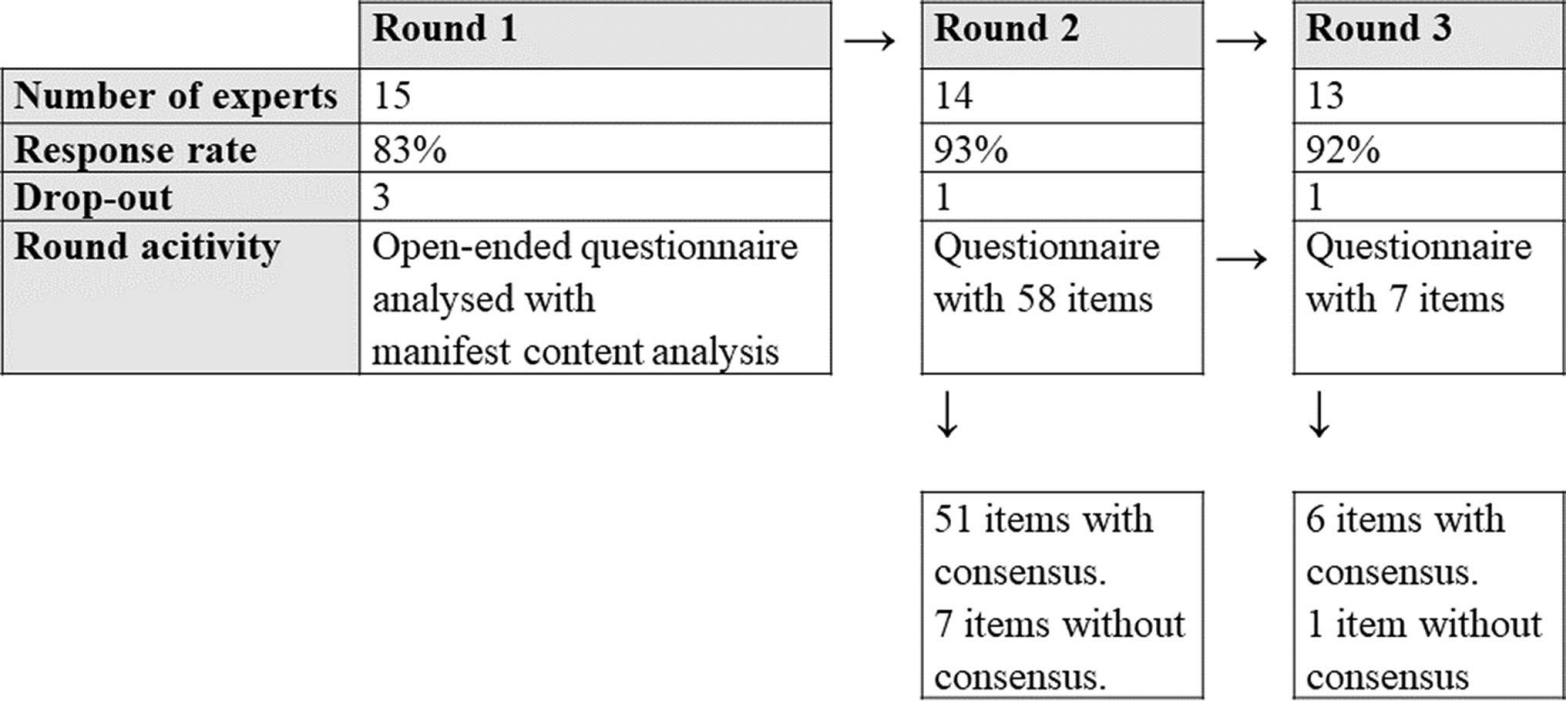
Flowchart of the study

The questionnaire developed in Round 1 was distributed online to the informants (n = 15), and three weeks were allowed for the responses. During this period, two reminder emails were sent to those who had not responded. In all, 14 informants answered the questionnaire in Round 2. The statements were reviewed in relation to the predefined consensus level of 75%. Before calculating the consensus level, the five-point Likert scale was trichotomized; 1–2 representing ‘disagree’, 3 represented ‘neutral’ and 4–5 represented ‘agree’. A statement was considered to have reached consensus when 75% or more of the informants agreed on any one of the trichotomized scales. In addition, for data analysis descriptive statistics was used. Mean value was used to rank the statements’ importance. Standard deviation and range were used to illustrate the eventual width within the group of informants in relation to each statement that reached consensus. The statements that reached consensus (n = 51) were removed from the questionnaire in the subsequent round (see Fig. 1).

#### Round 3

The revised questionnaire from round 2 was used for data collection in round 3. The statements that did not achieve consensus in round 2 (n = 7) were retained and distributed as per round 2. The questionnaire was accessible for 11 weeks as data collection occurred during the summer vacation period. Two reminders were sent to those who had not responded. In all, 13 informants took part in round 3. Consensus was determined using the same process as in round 2 along with descriptive statistics. In this round 6 additional items reached consensus, leaving 1 item with no consensus (see Fig. 1).

## Results

### Round 1

In the first round, 58 statements emerged from the content analysis, encompassing the three categories of knowledge (n = 26), skills (n = 14), and attitude (n = 18).

### Round 2–3

#### Knowledge

Twenty-six knowledge statements reached consensus (Table 2). The three statements with the highest mean were; ‘To care for people living with mental illness, knowledge of how to encounter the individual is needed’ (M:4.87 SD:0.35 R:4–5), ‘Knowledge of suicide processes (both suicide attempts and completed suicides) is needed to care for people living with mental illness’ (M:4.80 SD:0.42 R:4–5) and ‘Knowledge of how to counteract prejudice and stigma is needed to care for people living with mental illness’ (M:4.67 SD:0.62 R:3–5). The three statements with the lowest mean in this category were: ‘Knowledge of current legislation related to mental health care is needed to care for people living with mental illness’ (M:3.92 SD:0.76 R:2–5), ‘Knowledge of the organization of mental health care in municipalities and regions is needed to care for people living with mental illness’ (M:4.00 SD:0.93 R:2–5) and ‘Knowledge of pharmacological treatment is needed to care for people living with mental illness’ (M:4.07 SD:0.70 R: 3–5).

**Table 2:** Statements with consensus referring to ‘knowledge’ presented with Mean, Standard Deviation, Range, and in which round consensus was reached

Statement	Mean	SD^1^	Min.	Max.	Round^2^
To care for people living with mental illness, knowledge of how to encounter the individual is needed	4.87	0.35	4	5	2
Knowledge of suicide processes (both suicide attempts and completed suicides) is needed to care for people living with mental illness	4.80	0.42	4	5	2
Knowledge of how to counteract prejudice and stigma is needed to care for people living with mental illness	4.67	0.62	3	5	2
Knowledge of how people react in crisis situations is needed to care for people living with mental illness	4.67	0.49	4	5	2
Knowledge of comorbidity is needed to care for people living with mental illness	4.60	0.63	3	5	2
Knowledge of collaboration between different organisations is needed to care for people living with mental illness	4.60	0.51	4	5	2
Knowledge of how people can express themselves in, for example, psychosis or autism is needed to care for people living with mental illness	4.53	0.64	3	5	2
Knowledge in assessing the severity of the condition is needed to care for people living with mental illness	4.53	0.92	2	5	2
Knowledge about how to manage relatives is needed to care for people living with mental illness	4.47	0.83	2	5	2
Knowledge of psychiatric conditions is needed to care for people living with mental illness	4.47	0.64	3	5	2
Knowledge about caring for relatives—which includes children—is needed to care for people living with mental illness	4.47	0.64	3	5	2
Knowledge about self-harm is needed to care for people living with mental illness	4.47	0.52	4	5	2
Knowledge of where to get help within the healthcare organization is needed to care for people with living mental illness	4.40	0.83	2	5	2
Knowledge that there may be difficulties in describing one's situation and well-being is needed to care for people living with mental illness	4.40	0.74	3	5	2
Knowledge of communication methodology is needed to care for people living with mental illness	4.40	0.74	3	5	2
Knowledge of how the person's needs, resources and experiences should be appreciated is needed to care for people living with mental illness	4.33	0.62	3	5	2
Knowledge about inequalities is needed to care for people living with mental illness	4.31	0.63	3	5	3
Knowledge of diagnoses is needed to care for people living with mental illness	4.27	0.80	2	5	2
Knowledge about reduced functioning is needed to care for people living with mental illness	4.27	0.59	3	5	2
Knowledge about different types of addiction is needed to care for people living with mental illness	4.27	0.70	3	5	2
Knowledge about social vulnerability is needed to care for people living with mental illness	4.20	0.77	3	5	2
Knowledge of the side effects of pharmacological treatment is needed to care for people living with mental illness	4.13	0.64	3	5	2
Knowledge of supervision and debriefing is needed to care for people living with mental illness	4.08	0.64	3	5	3
Knowledge of pharmacological treatment is needed to care for people living with mental illness	4.07	0.70	3	5	2
Knowledge of the organization of mental health care in municipalities and regions is needed to care for people living with mental illness	4.00	0.93	2	5	2
Knowledge of current legislation related to mental health care is needed to care for people living with mental illness	3.92	0.76	2	5	3

1. Standard deviation (SD) 2. In which round consensus was reached

#### Skills

Thirteen skills statements reached consensus (Table 3). The three statements with the highest mean were: ‘Skills in dealing with anxiety and panic attacks are needed to be able to care for people living with mental illness’ (M:4.80 SD:0.41 R:4–5), ‘Sensitivity skills are needed to care for people living with mental illness’ (M: 4.73 SD:0.46 R:4–5) and ‘Communication skills are needed to care for people living with mental illness’ (M:4.73 SD:0.59 R: 3–5). The four statements with lowest mean were: ‘Skills in capturing the patient's understanding of what is sick or healthy are needed to care for people living with mental illness’ (M:4.23 SD:0.44 R:4–5). Three statements had the same mean (M:4.47), standard-deviation (SD:0.64) and range (R: 3–5): ‘Skills in identifying one's own ability/inability in emotionally difficult situations are needed to care for people living with mental illness’, ‘Skills in seeing what resources are around the patient (e.g. relatives) are needed to care for people living with mental illness’ and ‘Skills in translating theoretical knowledge about mental ill health into clinical interventions are needed to care for people living with mental illness’.

**Table 3:** Statements with consensus referring to ‘skills’ presented with Mean, Standard Deviation, Range and round in which consensus was reached

Statement	Mean	SD^1^	Min	Max	Round^2^
Skills in dealing with anxiety and panic attacks are needed to be able to care for people living with mental illness	4.80	0.41	4	5	2
Sensitivity skills are needed to care for people living with mental illness	4.73	0.46	4	5	2
Communication skills are needed to care for people living with mental illness	4.73	0.59	3	5	2
Skills in creating trustworthy encounters are needed to care for people living with mental illness	4.67	0.49	4	5	2
Skills in the use of non-verbal communication are needed to care for people living with mental illness	4.67	0.62	3	5	2
Skills in managing one's own emotions, even if you have met the patient many times before, are needed to treat people living with mental illness	4.60	0.63	3	5	2
Skills in assessing body language and other non-verbal cues are needed to care for people living with mental illness	4.53	0.52	4	5	2
Skills in talking about mental ill health, even in situations that may seem to be about physical illness, are needed to care for people living with mental illness	4.47	0.74	3	5	2
Skills in being able to adapt interventions according to the person's changing needs are needed to care for people living with mental illness	4.47	0.52	4	5	2
Skills in translating theoretical knowledge about mental ill health into clinical interventions are needed to care for people living with mental illness	4.47	0.64	3	5	2
Skills in seeing what resources are around the patient (e.g., relatives) are needed to care for people living with mental illness	4.47	0.64	3	5	2
Skills in identifying one's own ability/inability in emotionally difficult situations are needed to care for people living with mental illness	4.47	0.64	3	5	2
Skills in capturing the patient's understanding of what is sick or healthy are needed to care for people living with mental illness	4.23	0.44	4	5	3

1. Standard deviation (SD) 2. Round in which consensus was reached

#### Attitude

Eighteen attitudinal statements reached consensus (Table 4). The two statements with highest mean (M:4.80) had the same standard deviation (SD:0.41) and range (R:4–5): ‘Respectful behaviour is needed to care for people living with mental illness’ and ‘To be engaged during the consultation is needed to care for people living with mental illness’. Subsequent to this four statements presented with the same mean (M: 4.73), standard deviation (SD:0.46) and range (R:4–5): ‘A "non-judgmental" approach is needed to care for people living with mental illness’, ‘Responsiveness is needed to care for people living with mental illness’, ‘To remain calm in front of patients and others (e.g. relatives) is needed to care for people living with mental illness’ and ‘Be understanding of the fact that patients can be verbally aggressive because of their ill health is needed to care for people living with mental illness’. The three statements with lowest mean were: ‘A professional approach is needed to care for people living with mental illness’ (M:4.33 SD:0.82 R:2–5), ‘Questioning established working methods is needed to care for people living with mental illness’ (M:4.38 SD:0.65 R: 3–5) and ‘Being curious about what is happening right now is needed to care for people living with mental illness’ (M:4.38 SD:0.77 R: 3–5).

**Table 4:** Statements with consensus referring to ‘attitude’ presented with Mean, Standard Deviation, Range, and round in which consensus was reached

Statement	Mean	SD^1^	Min	Max	Round^2^
Respectful behaviour is needed to care for people living with mental illness	4.80	0.41	4	5	2
To be engaged during the consultation is needed to care for people living with mental illness	4.80	0.41	4	5	2
A "non-judgmental" approach is needed to care for people living with mental illness	4.73	0.46	4	5	2
Responsiveness is needed to care for people living with mental illness	4.73	0.46	4	5	2
To remain calm in front of patients and others (e.g., relatives) is needed to care for people living with mental illness	4.73	0.46	4	5	2
Be understanding of the fact that patients can be verbally aggressive because of their ill health is needed to care for people living with mental illness	4.73	0.46	4	5	2
Compassion is needed to care for people living with mental illness	4.67	0.49	4	5	2
Patience is needed to care for people living with mental illness	4.67	0.62	3	5	2
A genuine interest in the person is needed to care for people living with mental illness	4.67	0.62	3	5	2
A "low arousal" approach is needed to care for people living with mental illness	4.60	0.63	3	5	2
An empathetic attitude is needed to care for people living with mental illness	4.53	0.64	3	5	2
Dealing with one's own prejudices is needed to care for people living with mental illness	4.53	0.64	3	5	2
An open mind is needed to care for people living with mental illness	4.47	0.64	3	5	2
Affirmative behaviour is needed to care for people living with mental illness	4.47	0.83	3	5	2
A reflective approach is needed to care for people living with mental illness	4.47	0.74	3	5	2
Being curious about what is happening right now is needed to care for people living with mental illness	4.38	0.77	3	5	3
Questioning established working methods is needed to care for people living with mental illness	4.38	0.65	3	5	3
A professional approach is needed to care for people living with mental illness	4.33	0,82	2	5	2

1. Standard deviation (SD) 2. Round in which consensus was reached

## Discussion

To provide an overview of the statements that achieved consensus, the following headings are used for organizing the discussion: (1) *Counteracting prejudice and stigmatization* (e.g., statements such as "Knowledge of how to counteract prejudice and stigma is needed to care for people living with mental illness," "A 'non-judgmental' approach is needed to care for people living with mental illness," and "Respectful behavior is needed to care for people living with mental illness"); (2) *Acknowledging the person’s experience* (e.g., statements such as "To care for people living with mental illness, knowledge of how to engage with the individual is needed," "Sensitivity skills are needed to care for people living with mental illness," and "Responsiveness is needed to care for people living with mental illness"); and (3) *Identifying and assessing suicide risk* (e.g., statements such as "Knowledge of suicide processes, including suicide attempts and completed suicides, is needed to care for people living with mental illness").

### Counteracting prejudice and stigmatization

The results indicate that a required competence among ambulance clinicians is the capability to counter prejudice and stigmatization in the care of people living with mental illness. Being able to counter prejudice and stigmatization in people living with mental illness is important since this supports the creation of a safe and supportive care environment in which the person can feel comfortable. Research tells us that attitudes of ambulance clinicians towards people living with mental illness have not always been helpful since the clinicians felt unprepared due to limited training and stigmatization [34]. People living with mental illness are often exposed to prejudice and stigmatization from those around them [35]. This can, for instance, lead to people receiving poorer care and treatment [36]. Ambulance clinicians need to develop competence on how they can promote a helpful and respectful encounter with a person living with mental illness. This can be understood as being able to establish a caring encounter described as a reflective way of encompassing openness, sensitivity, empathy and ability to communicate appropriately [37]. Competence requires ambulance clinicians to demonstrate confidence, courage and professionalism, and showing respect and supporting dignity. This competence can also be a means to support ambulance clinicians in their efforts to reduce myths and stereotypes that exist about mental illness within ambulance care.

### Acknowledging the person’s experience

A competent ambulance clinician must be respectful and engage in the encounter utilising good communication skills and demonstrating sensitivity to the person’s perceived situation. During the encounter, ambulance clinicians need to adapt their care to meet person’s experience, needs, wishes and resources [38]. To be empathic is an essential competence in the encounter since it involves the capability to share feelings and create an environment of acceptance and understanding [39]. In the encounter, it is fundamental to create trust, confidence, and cooperation between the ambulance clinicians and the person living with mental illness [40]. A successful caring encounter may affect the person’s mental health by, for example, increasing self-esteem and confidence in one's own capabilities. However, research indicates that the experiences of people living with mental illness of ambulance care were dominated by misunderstandings and discrimination [41] which, in turn, can lead to a worsening of the person's suffering [42]. In the encounter it is important to be respectful of the individual. Being respectful includes, for example, how to behave, show consideration, being caring and acknowledging the person’s experiences, integrity, and self-determination [43]. Allowing autonomy to the individual is central to this respect as this ensures individuals are not forced to do anything they don't want to do, which might be perceived for them as problematic and challenging, for example, if the person has a pronounced conviction to commit suicide. This underlines the complex balance between ambulance clinicians’ responsibilities and supporting individuals' autonomy that can lead to conflicts and complex decisions [44]. It is also important to be present in the encounter and not to be distracted by other things. Being present is a way for the ambulance clinicians to show that they care about the person and that they want to help [45]. To be present is also about communication that includes the capability to listen actively, ask open and relevant questions, provide clear and tailored information, express respect, provide support and feedback, and handle sensitive and difficult situations [46]. When communication breaks down, there is a risk that several problems arise such as misunderstandings or a feeling that one is not heard or understood, which in turn influence the prerequisite for being sensitive to the person’s situation [47]. Consequently, it is important that ambulance clinicians develop their competence in establishing a caring encounter by being respectful and actively and openly listening to the person. Such competence should embrace skills to affirm the individuals’ feelings and experiences and provide them with support and hope even in situations where the encounter is characterized by tensions and conflicts.

### Identifying and assessing suicide risk

A required competence among ambulance clinicians is the ability to identify and assess suicide risk in people living with mental illness. Providing support to the individual is seen as an integrated component of this competence. Suicide is often the result of long-term and complex processes involving various risk factors, warning signs, crises, and decisions [48]. In managing people presenting with suicidal risk, it is necessary to know the factors that contribute to the suicide process, such as depression, anxiety, hopelessness, illness, or life events [49]. Understanding the suicide process is important to prevent suicide and provide adequate care and treatment to people who have suicidal thoughts or have attempted suicide [50]. Research indicates that ambulance clinicians could have an important part to play in preventing suicide, although suicidality is not always considered a task within the scope of ambulance care [51]. Such attitudes inhibit competence development by limiting access to information about available help, where to go for help, or how to comfort a person [52]. It can be assumed that this might be due to the attitudes and the occurrence of prejudice and stigmatization that exist within the ambulance service [35]. As suicidal behaviour is a life-threatening condition. Therefore, it is important that the ambulance clinicians have competence to react appropriately and take the necessary action in the encounter with patient who have suicidal thoughts or have attempted suicide. Ambulance clinicians also need to understand the role they can play in mitigating the risk of suicide and caring for people living with mental illness.

Finally, in ambulance care, all encounters with people are more or less spontaneous and it is important to be able to face individuals' care needs with openness to avoid routine and unreflective assessments of the person's condition and situation. The challenge in every encounter is to make an assessment embracing the person's perspective from the beginning [53]. To achieve this, the caring assessment and encounters needs to be highlighted and developed in both education and clinical practice. A caring assessment involves determining the appropriate nursing diagnoses based on the individual encounter with the person and encompasses relationships with the individual as well as attitudes and actions that promote people’s participation in their own care, with a view to ultimately take responsibility for their own health.

### Strength and limitations

This study used a Delphi method with the ambition to reach consensus within a panel of experts [31]. The panel members' varying backgrounds may be seen as a potential limitation. However, this can equally be viewed as a strength as caring in the ambulance context can be understood as a holistic practice having to take a broad range of perspectives into account [27]. It is unclear whether the experts' responses were based on situations in which ambulance clinicians are primarily required to conduct mental health assessments or in which mental health issues are comorbid with somatic concerns, which may constitute a limitation.

The size of the expert panel might be regarded as small. However, Delphi panels can vary from under 15 to 100 [31]. Literature tends to favour panel sizes between 8 and 50 participants and suggesting that panels with more than 30 participants seldom are found to improve results. A larger panel might have given other results, but also risked dropouts. Large dropout levels may have changed the overall characteristic of the expert panel altering the initial balance of the representation. Two experts dropped out during rounds 2 and 3. However, the reason for this is unknown due to ethical reasons, referring to the right to withdraw without providing an explanation.

The pre-defined level of consensus was set to 75% which can be deemed as high. There is no universal agreement in the literature regarding the appropriate level of consensus, and levels between 51 and 100% are supported [31]. However, the level of consensus depends on the research topic. We thought our 75% level of consensus would ensure the generation of statements that could be handled successfully while considering the transferability of the results into education and/or practice. The transferability of the findings may be considered through the efforts to describe, in detail, the participants, context, data collection, and analysis as carefully as possible. However, the study into this sparsely researched topic provides a first exploration and needs to be followed up with more research in other ambulance settings, nationally and internationally.

## Conclusion

This study has identified that the required competence in caring for people living with mental illness is about the capacity to counter prejudice and stigmatization, to acknowledge the person’s experience and to identify and assess suicide risk. Ambulance clinicians are expected to deal with a wide range of situations demanding caring assessments and clinically appropriate decisions. The foundation of a caring assessment is the establishment of a caring encounter with the individual in which a variety of competencies are demonstrated. To ensure that caring for people living with mental illness is adequate and appropriate, ambulance clinicians’ competencies must correspond to the requirements of their work. If education providers have an awareness of the required competency requirements, they can tailor the teaching and training of ambulance clinicians appropriately. Nevertheless, further research is needed to ensure that ambulance clinicians have the professional knowledge, skills, and attitudes needed to care for people living with mental illness.

## Supplementary Information

Below is the link to the electronic supplementary material.

Supplementary Material 1. The SRQR-checklist [32] is attached as supplementary material.

## Data Availability

The data used to support the findings of the study is not available due to ethical reasons.
