# Supplementary material for: Competence required while caring for people living with mental illness in the ambulance care setting: a Delphi study
Source: Discov Ment Health. 2025 Feb 21;5(1):17. doi: 10.1007/s44192-025-00140-6 (PMC11845652; doi:10.1007/s44192-025-00140-6)
Supplement: Supplementary file 1 — Supplementary Material 1. The SRQR-checklist [32] is attached as supplementary material. [file 44192_2025_140_MOESM1_ESM.docx]

| **Title and abstract** | | | |
| --- | --- | --- | --- |
| ***No.*** | ***Topic*** | ***Item*** | ***Authors’ assessment*** |
| S1 | Title | Concise description of the nature and topic of the study. Identifying the study as qualitative or quantitative, indicating the approach (e.g., ethnography, grounded theory) or data collection methods (e.g., interview, focus group) are recommended. | Yes, see title. |
| S2 | Abstract | Summary of key elements of the study using the abstract format of the intended publication; typically includes background, purpose, methods, results, and conclusions. | Yes, see abstract. |
| **Introduction** | | | |
| ***No.*** | ***Topic*** | ***Item*** | ***Authors’ assessment*** |
| S3 | Problem formulation | Description and significance of the problem/phenomenon studied; review of relevant theory and empirical work; problem statement. | Yes, see under the heading ‘Introduction’. |
| S4 | Purpose of research question | Purpose of the study and specific objectives or questions. | Yes, see under the heading ‘Aim’. |
| **Methods** | | | |
| ***No.*** | ***Topic*** | ***Item*** | ***Authors’ assessment*** |
| S5 | Qualitative approach and research paradigm | Qualitative approach (e.g., ethnography, grounded theory, case study, phenomenology, narrative research) and guiding theory if appropriate;  identifying the research paradigm (e.g., postpositivist, constructivist/interpretivist) is also recommended. | Yes, see under the heading ‘Materials and methods’. |
| S6 | Researchers’ characteristics and reflexivity | Researchers’ characteristics that may influence the research, including personal attributes, qualifications/experience, relationship with participants, assumptions, and/or presuppositions; potential or actual interaction between researchers’ characteristics and the research questions, approach, methods, results, and/or transferability | Yes, see under the heading ‘Data collection and analysis’. |
| S7 | Context | Setting/site and salient contextual factors; rationale. | Yes, se under the heading ‘Expert panel’. |
| S8 | Sampling strategy | How and why research participants, documents, or events were selected; criteria for deciding when no further sampling was necessary (e.g., sampling saturation). | Yes, see under the headings ‘Expert panel’ and ‘Strength and limitations’. |
| S9 | Ethical issues pertaining to human subjects | Documentation of approval by an appropriate ethics review board and participant consent, or explanation for lack thereof; other confidentiality and data security issues. | Yes, see under the heading ‘Ethical Approval’. |
| S10 | Data collection methods | Types of data collected; details of data collection procedures including (as appropriate) start and stop dates of data collection and analysis, iterative process, triangulation of sources/methods, and modification of procedures in response to evolving study findings. | Yes, see under the heading ‘Data collection and analysis’. |
| S11 | Data collection instruments and technologies | Description of instruments (e.g., interview guides, questionnaires) and devices (e.g., audio recorders) used for data collection; if/how the instrument(s) changed over the course of the study. | Yes, see under the heading ‘Data collection and analysis’. |
| S12 | Units of study | Number and relevant characteristics of participants, documents, or events included in the study; level of participation (could be reported in results). | Yes, see under the heading ‘Expert panel’ and ‘Strengths and limitations’ together with Table 1 and Figure 1. |
| S13 | Data processing | Methods for processing data prior to and during analysis, including transcription, data entry, data management and security, verification of data integrity, data coding, and anonymization/deidentification of excerpts. | Yes, see under the heading ‘Data collection and analysis’ (Round 1 to 3). |
| S14 | Data analysis | Process by which inferences, themes, etc., were identified and developed, including the researchers involved in data analysis; usually references a specific paradigm or approach. | Yes, see under the heading ‘Data collection and analysis’ (Round 1 to 3). |
| S15 | Techniques to enhance trustworthiness | Techniques to enhance trustworthiness and credibility of data analysis (e.g., member checking, audit trail, triangulation). | Yes, this lies in the method itself as the expert panels in the questionnaire consider and value statements emerging from their own free text answers, see under the heading ‘Data collection and analysis’. |
| **Results/findings** | | | |
| ***No.*** | ***Topic*** | ***Item*** | ***Authors’ assessment*** |
| S16 | Synthesis and intepretation | Main findings (e.g., interpretations, inferences, and themes); might include development of a theory or model, or integration with prior research or theory. | Yes, see under the heading ‘Results’. |
| S17 | Links to empirical data | Evidence (e.g., quotes, field notes, text excerpts, photographs) to substantiate analytic findings. | Yes, see under the heading ‘Data collection and analysis’ and ‘Results’. |
| **Discussion** | | | |
| ***No.*** | ***Topic*** | ***Item*** | ***Authors’ assessment*** |
| S18 | Integration with prior work, implications, transferability, and contribution(s) to the field | Short summary of main findings; explanation of how findings and conclusions connect to, support, elaborate on, or challenge conclusions of earlier scholarship; discussion of scope of application/generalizability; identification of unique contribution(s) to scholarship in a discipline or field. | Yes, see under the heading ‘Discussion’. |
| S19 | Limitations | Trustworthiness and limitations of findings. | Yes, see under the heading ‘Strengths and limitations’. |
| **Other** | | | |
| ***No.*** | ***Topic*** | ***Item*** | ***Authors’ assessment*** |
| S20 | Conflict of interest | Potential sources of influence or perceived influence on study conduct and conclusions; how these were managed. | Yes, no conflict of interest is declared under the heading ‘Conflict of interest’. |
| S21 | Funding | Sources of funding and other support; role of funders in data collection, interpretation, and reporting. | Yes, see under the heading ‘Funding’. |

1. O’Brien BC, Harris, IB, Beckman TJ, Reed DA, Cook DA. Standards for Reporting Qualitative Research: A Synthesis of Recommendations. Academic Medicine. 2014;89:1245-1251.
